# Donor Human Milk Use in Advanced Neonatal Care Units — United States, 2020

**DOI:** 10.15585/mmwr.mm7133a1

**Published:** 2022-08-19

**Authors:** Ellen O. Boundy, Erica H. Anstey, Jennifer M. Nelson

**Affiliations:** 1 Division of Nutrition, Physical Activity, and Obesity, National Center for Chronic Disease Prevention and Health Promotion, CDC.

Approximately 50,000 infants are born in the United States each year with very low birthweight (VLBW) (<1,500 g).* Benefits of human milk to infants with VLBW include decreased risk for necrotizing enterocolitis, a serious illness resulting from inflammation and death of intestinal tissue that occurs most often in premature infants, especially those who are fed formula rather than human milk; late-onset sepsis; chronic lung disease; retinopathy of prematurity; and neurodevelopmental impairment (*1*). When mother’s own milk is unavailable or insufficient, pasteurized donor human milk (donor milk) plus a multinutrient fortifier is the first recommended alternative for infants with VLBW (*2*). CDC’s 2020 Maternity Practices in Infant Nutrition and Care (mPINC) survey was used to assess practices for donor milk use in U.S. advanced neonatal care units of hospitals that provide maternity care (*3*). Among 616 hospitals with neonatal intensive care units (level III or IV units),^†^ 13.0% reported that donor milk was not available for infants with VLBW; however, approximately one half (54.7%) reported that most (≥80%) infants with VLBW do receive donor milk. Donor milk availability for infants with VLBW was more commonly reported among hospitals with a level IV unit, higher annual birth volume, location in the Midwest and Southwest regions, nonprofit and teaching status, and those designated Baby-Friendly.^§^ Addressing hospitals’ barriers to providing donor milk could help ensure that infants with VLBW receive donor milk when needed and help reduce morbidity and mortality in infants with VLBW (*1*,*4*).

The mPINC survey is a biennial census of all maternity care hospitals in the United States and territories to monitor practices and policies related to infant feeding. The survey is completed electronically by the persons most knowledgeable about the hospital’s practices related to infant nutrition. In 2020, hospitals with advanced neonatal care units (level II, III, or IV) were asked how many infants (<1,500 g and ≥1,500 g) receive donor milk at any time while in the unit: few (0%–19%), some (20%–49%), many (50%–79%), most (≥80%), or donor milk not available.

The prevalence of donor milk use was examined by unit level and infant weight^¶^ (<1,500 g and ≥1,500 g). For infants weighing ≥1,500 g, analyses included hospitals with level II, III, or IV units. Analyses for infants weighing <1,500 g were restricted to hospitals with level III or IV units, where infants with VLBW typically receive care (*3*). Donor milk use among infants with VLBW was also examined by hospital characteristics: hospital type, teaching hospital status, Baby-Friendly designation, number of annual births, and region.** Availability was also examined by state or territory (state) by calculating the percentage of participating hospitals with a level III or IV neonatal intensive care unit in each state reporting that donor milk was available for infants with VLBW. Data were suppressed for states with fewer than five hospitals reporting. Descriptive analyses were conducted using SAS (version 9.4; SAS Institute). Because this is a census sample, SEs were not calculated, and statistical testing was not performed. This activity was reviewed by CDC and was conducted consistent with applicable federal law and CDC policy.^††^

In 2020, among 2,810 eligible maternity hospitals, 2,103 (74.8%) participated in mPINC. Among participating hospitals, 1,260 (59.9%) reported having an advanced neonatal care unit, including 642 (60.0%) level II, 528 (41.9%) level III, and 90 (7.1%) level IV units. Hospitals that did not answer the donor milk question were excluded, resulting in analytic samples of 616 hospitals with level III and IV units for infants <1,500 g and 1,256 hospitals with level II, III, or IV units for infants ≥1,500 g.

Among hospitals with level III or IV units, 13.0% reported that donor milk was not available for infants with VLBW, and 54.7% reported it was received by ≥80% of infants with VLBW (Table 1). Among hospitals with level II, III, or IV units, for infants weighing ≥1,500 g, 40.1% reported that donor milk was not available, and 15.9% reported that it was received by most of these infants. For both weight categories, donor milk was more commonly available and used at hospitals with level IV units than in those with level II or III.

**TABLE 1:** Donor milk use among infants in hospitals with advanced neonatal care units, by infant weight and unit level — Maternity Practices in Infant Nutrition and Care, United States, 2020*^,†^

Infant weight/Neonatal care unit level	No. of hospitals	% of hospitals^§,¶^				
		Donor milk not available	% of infants receiving donor milk			
			0–19	20–49	50–79	≥80
**<1,500 g**						
**Total**	**616**	**13.0**	**5.0**	**10.1**	**17.2**	**54.7**
Level III	526	14.8	4.4	9.9	17.1	53.8
Level IV	90	2.2	8.9	11.1	17.8	60.0
**≥1,500 g**						
**Total**	**1,256**	**40.1**	**14.7**	**14.7**	**14.6**	**15.9**
Level II	640	65.3	7.0	7.3	8.0	12.3
Level III	526	15.8	23.4	21.7	20.2	19.0
Level IV	90	3.3	17.8	26.7	28.9	23.3

* SEs were not calculated, and statistical testing not performed, because Maternity Practices in Infant Nutrition and Care is a census sample.

^†^ Level II = special care nursery; level III = neonatal intensive care unit; level IV = regional neonatal intensive care unit. https://doi.org/10.1542/peds.2012-1999

^§^ Hospitals reporting the percentage of infants who receive donor human milk at any time while cared for in the advanced neonatal care unit.

^¶^ Row percentages might not sum to 100% because of rounding.

Donor milk was reported to be unavailable for infants with VLBW in 11.6% of nonprofit, 16.0% of for-profit, and 17.1% of government or military hospitals (Table 2). Among teaching hospitals, 12.4% reported that donor milk was not available, and 53.3% reported it was received by ≥80% of infants with VLBW, compared with 16.9% and 64.0%, respectively, among nonteaching hospitals. Donor milk was not available for infants with VLBW in 11.1% of Baby-Friendly designated hospitals, compared with 14.3% of non–Baby-Friendly designated hospitals. Although donor milk was available for infants with VLBW in almost all (97.8%) level IV units (Table 1), its availability and use among hospitals with a level III unit varied by hospital size. Among the largest hospitals with a level III unit (≥5,000 annual births), 6.3% reported that donor milk was not available, and 40.6% reported it was received by ≥80% of infants with VLBW, compared with 44.0% and 36.0%, respectively, among the smallest such hospitals (<1,000 annual births). By region, nonavailability of donor milk for infants with VLBW ranged from 4.1% of hospitals in the Midwest to 23.8% in the Northeast, among those with level III or IV units.

**TABLE 2:** Donor milk use among infants weighing <1,500 g in hospitals with a level III or IV neonatal intensive care unit, by hospital characteristics — Maternity Practices in Infant Nutrition and Care, United States, 2020*^,†^

Characteristic	No. of hospitals	% of hospitals^§,¶^				
		Donor milk not available	% of infants receiving donor milk			
			0–19	20–49	50–79	≥80
**Total**	**616**	**13.0**	**5.0**	**10.1**	**17.2**	**54.7**
**Hospital type**						
Nonprofit, private	438	11.6	5.5	9.4	17.4	56.2
For-profit, private	94	16.0	3.2	10.6	14.9	55.3
Government or military	82	17.1	4.9	12.2	18.3	47.6
**Teaching hospital status**						
Yes	525	12.4	5.7	10.7	17.9	53.3
No	89	16.9	1.1	5.6	12.4	64.0
Baby-Friendly** hospital designation						
Yes	244	11.1	4.9	11.5	17.2	55.3
No	370	14.3	5.1	8.9	17.0	54.6
**Annual no. of live births**						
<1,000	53	41.5	5.7	5.7	9.4	37.3
1,000–1,999	201	13.9	4.5	7.0	15.4	59.2
2,000–4,999	315	8.9	5.1	11.1	18.4	56.5
≥5,000	47	4.3	6.4	21.3	25.5	42.6
**Region^††^**						
Midwest	97	4.1	5.2	12.4	20.6	57.7
Southwest	111	6.3	4.5	10.8	16.2	62.2
Mid-Atlantic	89	13.5	4.5	7.9	22.5	51.7
Southeast	102	13.7	4.9	15.7	17.7	48.0
Mountain Plains	50	16.0	6.0	12.0	8.0	58.0
Western	104	19.2	5.8	2.9	12.5	59.6
Northeast	63	23.8	4.8	9.5	20.6	41.3

* SEs were not calculated, and statistical testing not performed, because Maternity Practices in Infant Nutrition and Care is a census sample.

^†^ Level II = special care unit; level III = neonatal intensive care unit; level IV = regional neonatal intensive care unit. https://doi.org/10.1542/peds.2012-1999

^§^ Hospitals reporting the percentage of infants weighing <1,500 g who receive donor human milk at any time while cared for in a neonatal intensive care unit.

^¶^ Row percentages might not sum to 100% because of rounding.

** Baby-Friendly USA is the accrediting body and national authority for the Baby-Friendly Hospital Initiative (BFHI) in the United States. BFHI is a global program to encourage the broad-scale implementation of steps to provide mothers with information, confidence, and skills necessary to successfully initiate and continue breastfeeding. https://www.babyfriendlyusa.org

^††^ Regions defined by U.S. Department of Agriculture Food and Nutrition Service. https://www.fns.usda.gov/fns-regional-offices

Twenty-three U.S. states had at least 10 hospitals with a level III or IV neonatal intensive care unit, 13 had five to nine level III or IV hospitals, 15 had one to four level III or IV hospitals, and five had no hospital with level III or IV neonatal intensive care units participating in mPINC. Among the 36 states with five or more hospitals with a level III or IV unit, the statewide percentage of hospitals reporting donor milk availability for infants with VLBW ranged from 0% to 100% (median = 92.0%) (Figure). In 12 states (Alabama, Arkansas, Colorado, Indiana, Iowa, Massachusetts, Minnesota, New Mexico, Oregon, Utah, Washington, and Wisconsin), 100% of hospitals with level III or IV units reported donor milk was available for infants with VLBW; in seven states (Illinois, Maryland, North Carolina, Ohio, Pennsylvania, Texas, and Virginia), 90% to <100% of hospitals reported donor milk availability; in 10 states (Connecticut, Florida, Kentucky, Louisiana, Michigan, Mississippi, Nebraska, New Jersey, South Carolina, and Tennessee), 80% to <90% of hospitals reported donor milk availability; and in seven jurisdictions (California, Georgia, Kansas, Missouri, New York, Oklahoma, and Puerto Rico), <80% of hospitals reported that donor milk was available.

**FIGURE:**
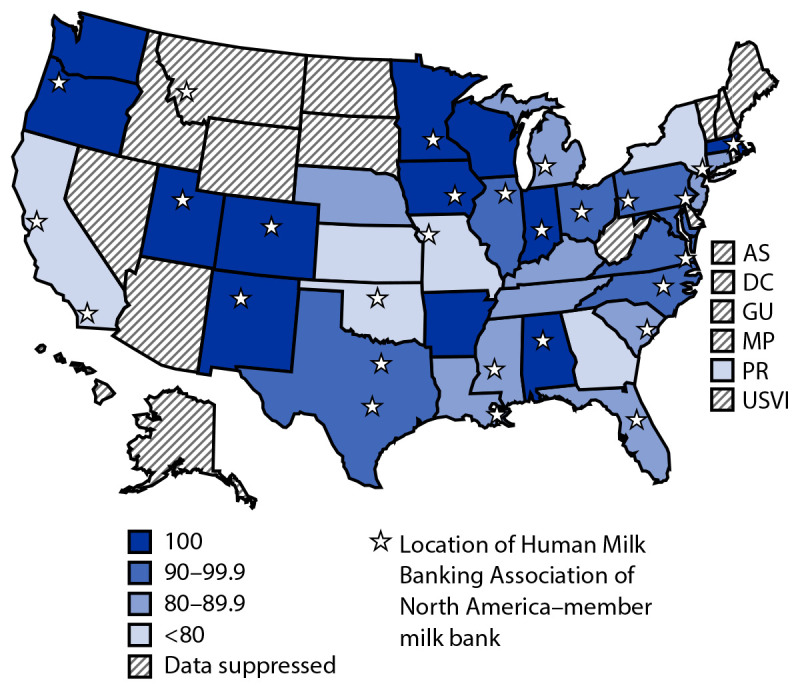
Percentage of hospitals with level III or IV neonatal intensive care units reporting donor milk was available for infants weighing <1,500 g, by state* — Maternity Practices in Infant Nutrition and Care, United States, 2020 **Abbreviations:** AS = American Samoa; DC = District of Columbia; GU = Guam; MP = Northern Mariana Islands; PR = Puerto Rico; USVI = U.S. Virgin Islands. * Includes all U.S. states, territories, and DC; data were suppressed when the sample was <5. The locations of 28 Human Milk Banking Association of North America–member milk banks are also noted.

## Discussion

Although human milk is the recommended nutrition source for infants with VLBW, with donor milk as the preferred alternative to mother’s own milk when needed, this analysis found that donor milk was unavailable or not frequently used in some hospitals caring for those infants. In mPINC 2020, 13.0% of hospitals with a level III or IV unit reported donor milk was not available for infants with VLBW; however, availability might be improving. In CDC’s 2018 mPINC survey, 16.5% of hospitals with a level III or IV unit reported donor milk was not available for infants with VLBW (CDC, unpublished data, 2022). In general, availability and use of donor milk for infants in advanced care units appears to be increasing over time. A 2011 study using mPINC data found that 45.2% of U.S. hospitals with a neonatal intensive care unit reported ever using donor milk (for infants of any birthweight); an increase from 25.1% in 2007 and 28.7% in 2009 (*5*).

Limitations in the availability and use of donor milk for infants with VLBW might be due to a variety of factors. Most hospitals access donor milk from banks accredited by the nonprofit Human Milk Banking Association of North America, with 28 member milk banks currently operating in 25 states.^§§^ Availability of donor milk at hospitals might be affected by supply from milk banks, cost, and reimbursement, which can vary by state and payment source (*6*). Milk bank supply is in turn affected by barriers persons might face when considering milk donation, such as lack of knowledge about milk banking and beliefs about acceptability of donation (*7*). Hospital leadership support and logistical challenges to implementing donor milk programs might also play a role in donor milk availability (*8*).

When donor milk is available, additional hospital- and individual-level factors might affect how often it is used. These include lack of standardized policies and staff member training related to donor milk use, as well as staff member and parent knowledge and perceptions about the health benefits and safety of donor milk (*6*).

The findings in this report are subject to at least three limitations. First, the percentage of infants with VLBW needing supplementation to mother’s own milk or full feedings with donor milk is not well documented, making interpreting prevalence estimates of donor milk use among hospitals where it is available challenging because the ideal prevalence is not known. Second, there is potential for social desirability bias or other measurement error because hospitals’ use of donor milk is self-reported. Finally, mPINC does not collect data from neonatal units in hospitals that do not provide maternity care, such as children’s hospitals; therefore, donor milk use for infants with VLBW in those settings is not represented in this analysis.

Addressing barriers related to the availability of milk banks, donation to milk banks, use of donor milk in hospitals, and knowledge and attitudes about donor milk could potentially increase its availability and use for infants with VLBW. The American Academy of Pediatrics and Baby-Friendly USA recently published documents outlining recommended practices for promoting human milk use for infants with VLBW and in the neonatal intensive care setting, which could provide guidance to hospitals implementing a donor milk program (*1*,*4*). State Perinatal Quality Collaboratives are another tool that could help birthing hospitals implement quality improvement initiatives to increase access to and use of donor milk to reduce morbidity and mortality in infants with VLBW.^¶¶^

**Summary**

**What is already known about this topic?**

Infants with very low birthweight (VLBW) are at increased risk for long- and short-term health problems. Human milk is the recommended nutrition source for infants with VLBW, who should receive supplemental donor milk when mother’s own milk is insufficient or unavailable.

**What is added by this report?**

Analysis of CDC’s 2020 Maternity Practices in Infant Nutrition and Care survey data found that donor milk was not available for infants with VLBW at 13.0% of U.S. hospitals with neonatal intensive care units (level III or IV).

**What are the implications for public health practice?**

Identifying and addressing barriers to provision of donor milk for infants with VLBW could help ensure that these infants receive donor milk when needed and help decrease associated morbidity and mortality.

## Footnotes

*
https://www.cdc.gov/nchs/data/nvsr/nvsr70/nvsr70-02-tables-508.pdf

† Level II = special care nursery; level III = neonatal intensive care unit; level IV = regional neonatal intensive care unit. https://doi.org/10.1542/peds.2012-1999

§ Baby-Friendly USA is the accrediting body and national authority for the Baby-Friendly Hospital Initiative (BFHI) in the United States. BFHI is a global program to encourage the broad-scale implementation of steps to provide mothers with information, confidence, and skills necessary to successfully initiate and continue breastfeeding. https://www.babyfriendlyusa.org

¶ In response to the survey question “How many infants receive donor human milk at any time while cared for in your hospital’s Special Care Nursery (level II)/Neonatal Intensive Care Unit (levels III, IV)? Infants <1500 grams, infants ≥1500 grams.”

**
https://www.fns.usda.gov/fns-regional-offices#

†† 45 C.F.R. part 46, 21 C.F.R. part 56; 42 U.S.C. Sect. 241(d); 5 U.S.C. Sect. 552a; 44 U.S.C. Sect. 3501 et seq.

§§
https://www.hmbana.org/find-a-milk-bank/overview.html

¶¶
https://www.cdc.gov/reproductivehealth/maternalinfanthealth/pqc.htm

## References

[1] Parker MG, Stellwagen LM, Noble L, Kim JH, Poindexter BB, Puopolo KM; Section on Breastfeeding; Committee on Nutrition; Committee on Fetus and Newborn. Promoting human milk and breastfeeding for the very low birth weight infant. Pediatrics 2021;148:e2021054272. 10.1542/peds.2021-054272 34635582

[2] Daniels S, Corkins M, de Ferranti S, ; Committee on Nutrition; Section on Breastfeeding; Committee on Fetus and Newborn. Donor human milk for the high-risk infant: preparation, safety, and usage options in the United States. Pediatrics 2017;139:e20163440. 10.1542/peds.2016-3440 27994111

[3] Barfield WD, Papile L-A, Baley JE, ; American Academy of Pediatrics Committee on Fetus and Newborn. Levels of neonatal care. Pediatrics 2012;130:587–97. 10.1542/peds.2012-1999 22926177

[4] Baby-Friendly USA. Inc. Neonatal intensive care (NICU) resources: a guide to recommended practices. Albany, NY: Baby-Friendly USA; 2021. https://www.babyfriendlyusa.org/wp-content/uploads/2021/08/BFUSA-NICU-Resources.pdf

[5] Perrine CG, Scanlon KS. Prevalence of use of human milk in US advanced care neonatal units. Pediatrics 2013;131:1066–71. 10.1542/peds.2012-3823 23669517 PMC4535053

[6] Bai Y, Kuscin J. The current state of donor human milk use and practice. J Midwifery Womens Health 2021;66:478–85. 10.1111/jmwh.13244 34250723

[7] Doshmangir L, Naghshi M, Khabiri R. Factors influencing donations to human milk bank: a systematic review of facilitators and barriers. Breastfeed Med 2019;14:298–306. 10.1089/bfm.2019.0002 30896254

[8] Rosenbaum K. Implementing the use of donor milk in the hospital setting: implications for nurses. Nurs Womens Health 2012;16:202–8. 10.1111/j.1751-486X.2012.01731.x 22697223

